# ARE WE GOING TO FEEL COMFORTABLE THERE?: EMERGENT FINDINGS FROM THE DIVERSITY IN LIFE PLAN COMMUNITIES STUDY

**DOI:** 10.1093/geroni/igac059.2941

**Published:** 2022-12-20

**Authors:** Mushira Khan, Pankaja Desai, Catherine O’Brien

**Affiliations:** Mather Institute, Evanston, Illinois, United States; Rush University Medical Center, Chicago, Illinois, United States; Mather, Evanston, Illinois, United States

## Abstract

Life Plan Communities (LPCs), formerly known as Continuing Care Retirement Communities, offer an array of amenities as well as a continuum of care where residents may live independently or access assisted living or skilled care if needed. Racial/ethnic minority older adults are under-represented among LPC residents. One likely contributor to this lack of diversity is that racial/ethnic minorities make up a relatively small proportion of higher income groups. However, there may be additional reasons for their relative absence in most LPCs. The purpose of the ‘Diversity in Life Plan Communities’ study is to (1) identify barriers that impact the decision to move into an LPC for racial/ethnic minorities, and (2) identify strategies to increase resident diversity in LPCs. In this presentation we share preliminary findings from in-depth, semi-structured, qualitative interviews with community-dwelling Black, Latinx, and South Asian adults (n=10) living in Chicagoland and case study interviews with current LPC ‘resident champions’ (n=3) who are actively involved in efforts to increase resident diversity at their respective LPCs. Thematic analysis of the data showed that lack of awareness about LPCs, concerns about racism, desire to age-in-place among loved ones, and perceived non-availability of culturally-congruent activities were key barriers to moving into an LPC. These findings suggest that in order to increase resident diversity within their communities, LPC operators, staff, and current residents may consider targeted outreach efforts in racial/ethnic communities, take steps to create a welcoming environment and engage in more culturally congruent activities, and involve adult children/grandchildren in the decision-making process.

